# Intestinal Perforation due to Neutropenic Enterocolitis in a Patient Treated with Bevacizumab for Ovarian Cancer

**DOI:** 10.1155/2020/7231358

**Published:** 2020-06-16

**Authors:** Akinori Sasaki, Kenichi Harano, Takahiro Kogawa, Nobuaki Matsubara, Yoichi Naito, Ako Hosono, Hirofumi Mukai, Takayuki Yoshino, Toru Mukohara

**Affiliations:** ^1^Department of Breast and Medical Oncology, National Cancer Center Hospital East, Kashiwa, Chiba, Japan; ^2^Department of Gastroenterology and Gastrointestinal Oncology, National Cancer Center Hospital East, Kashiwa, Chiba, Japan

## Abstract

Intestinal perforation is a rare adverse event of antineoplastic therapy. However, once it occurs, it is potentially fatal. This report describes a case of intestinal perforation caused by bevacizumab in a patient with ovarian cancer who concurrently developed neutropenic enterocolitis. A 66-year-old woman diagnosed with metastatic ovarian cancer received combination chemotherapy with carboplatin, gemcitabine, and bevacizumab. On day 14, she developed grade 4 pancytopenia and febrile neutropenia, which resulted in neutropenic enterocolitis and intestinal perforation. Emergency surgery was performed, and an intestinal perforation found in the ascending colon was closed. Postoperatively, she developed an intra-abdominal abscess requiring peritoneal drainage. She was discharged from the hospital on recovery.

## 1. Introduction

Vascular endothelial growth factor (VEGF) A is a proangiogenic growth factor, which stimulates the proliferation, migration, and survival of endothelial cells. VEGF also plays a role in tumor growth and is the key target of certain antitumor drugs [1]. Bevacizumab, a humanized anti-VEGF monoclonal IgG_1_ antibody, inhibits neovascularization in tumor tissue and reduces the pressure of the tumor tissue framework [2]. The efficacy of bevacizumab has been proven in colorectal, breast, ovarian, and non-small-cell lung cancers [3–6]. Gastrointestinal perforation, known to be potentially fatal, is a rare adverse effect of bevacizumab therapy. Although neutropenic enterocolitis occurs in patients with neutropenia, it rarely leads to intestinal perforation [7]. Surgery for intestinal perforation carries high risk in patients with neutropenic enterocolitis owing to associated neutropenia and thrombocytopenia.

This report describes our experience with a patient who developed intestinal perforation due to the combined effect of neutropenic enterocolitis and bevacizumab.

## 2. Case Presentation

A 66-year-old Japanese woman presented with symptoms of abdominal distension and anorexia and was diagnosed with ovarian cancer (clear cell carcinoma), stage IIIC. She received neoadjuvant combination chemotherapy with carboplatin (AUC 5, day 1, every 3 weeks) and paclitaxel (175 mg/m^2^, day 1, every 3 weeks). She experienced grade 2 neutropenia during the first cycle; however, she recovered from this adverse event without the complication of infections. In addition, she achieved partial response in CT after two cycles of chemotherapy. Interval debulking surgery (IDS) was performed after seven cycles of this chemotherapy. Total hysterectomy, salpingo-oophorectomy, and infracolic omentectomy were performed for this patient. No major postoperative complications were observed in this case. On recovery, she received two cycles of adjuvant chemotherapy. Despite achieving complete response following treatment, she presented with recurrent peritoneal dissemination of the tumor, seven months after the last chemotherapy cycle. She was diagnosed with platinum-sensitive relapsed ovarian cancer and was prescribed combination chemotherapy with carboplatin (AUC 4, day 1, every 3 weeks), gemcitabine (1000 mg/m^2^, days 1 and 8, every 3 weeks), and bevacizumab (15 mg/kg, day 1, every 3 weeks).

**Figure 1:**
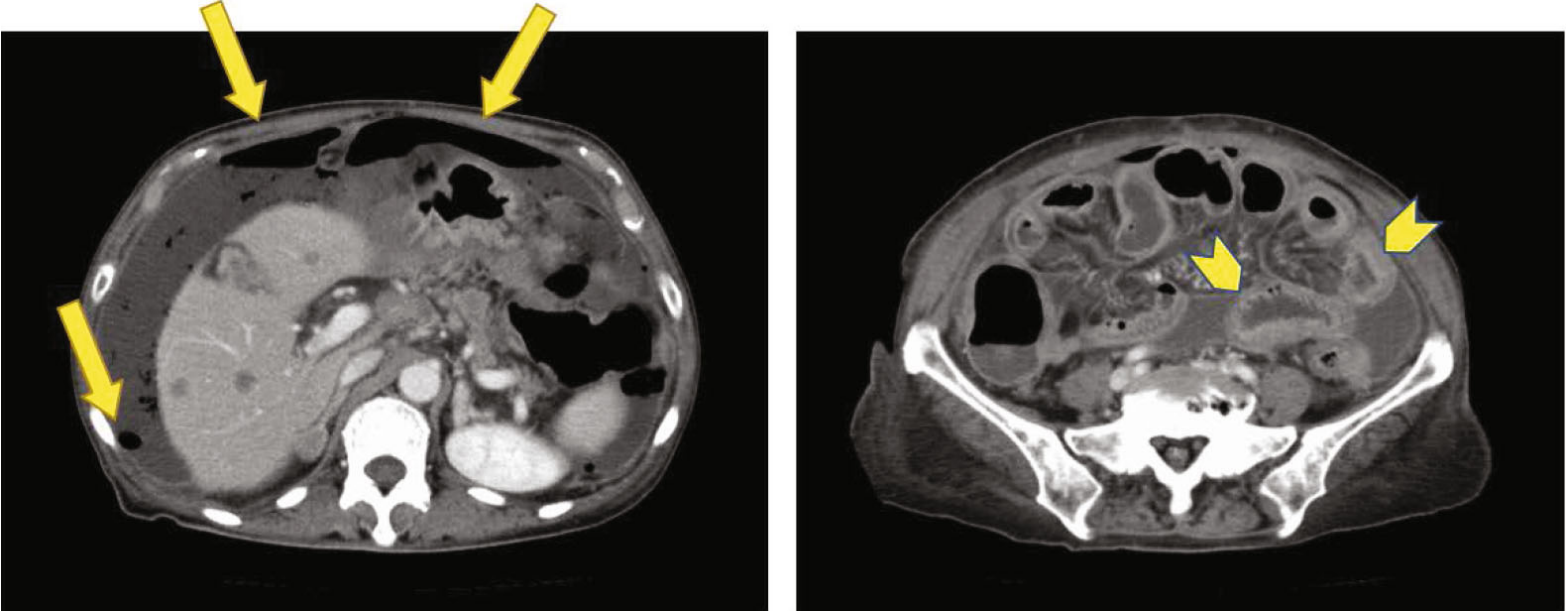
The abdominal CT findings. Abdominal CT showed free air (single arrow) and thickening of the bowel wall (arrowhead).

She did not experience any adverse events for several days after administration of second-line chemotherapy. However, on day 14 of the first cycle, she presented to the hospital with fever and was subsequently diagnosed with febrile neutropenia owing to severe reductions in absolute neutrophil counts, which was evident from the laboratory data (Table 1). She also had thrombocytopenia of grade 4 and was suspected to have neutropenic enterocolitis owing to the presence of nausea and watery diarrhea, without any abdominal pain. After admission to the hospital, she received platelet transfusions and antibiotics, in addition to granulocyte colony-stimulating factor (G-CSF). On day 17, she complained of acute abdominal pain. The whole abdomen was tender on palpation, and rebound tenderness was elicited. Computed tomography was performed, which demonstrated thickening of the bowel wall with gastrointestinal perforation (Figure 1). She then underwent emergency surgery. Intraoperatively, the peritoneal cavity revealed turbid ascitic fluid exceeding 1000 mL in volume, with numerous white nodules characteristic of peritoneal dissemination. The entire intestine was markedly edematous, fragile, and inflamed, suggestive of neutropenic enterocolitis. The perforation site, which was markedly edematous, was detected in the ascending colon. A closure of the perforation was performed with placement of an intraperitoneal drain. The postoperative period was complicated with the development of an intra-abdominal abscess, requiring the placement of an additional drain, with appropriate antibiotics. Her condition improved gradually, and she was discharged from the hospital on day 56 after a complete recovery.

## 3. Discussion

In the present case, intestinal perforation was induced by bevacizumab in the presence of neutropenic enterocolitis. Intestinal perforation is a fatal condition, particularly in neutropenic patients; urgent surgical intervention is essential for its management. Prompt assessment and formulation of a management plan are key factors in improving patient outcomes. In the present case, treatment of the intestinal perforation was successful owing to early detection and treatment.

Bevacizumab, a humanized anti-VEGF monoclonal IgG_1_ antibody, has demonstrated clinical benefit in ovarian cancer and has been available in Japan since 2013. Although bevacizumab shows considerable benefits, several adverse events have been reported with its use. Gastrointestinal adverse events are common with bevacizumab, and nausea, anorexia, and constipation are frequent. However, gastrointestinal perforation is a rare event, occurring in 0.9% to 3% of these patients [8–10]; it leads to severe peritonitis, which is a potentially fatal adverse event, with mortality rates exceeding 20% [11]. Bevacizumab-induced gastrointestinal perforations may be caused by various mechanisms. A previous study had speculated that bevacizumab possibly damages the structural and functional integrity of the gastrointestinal vasculature, causing ischemic perforation [12]. A report suggested that bevacizumab prevents healing of intestinal inflammation and ulcers, which leads to subsequent perforations [13].

Several risk factors predispose patients to develop gastrointestinal perforation. Medical conditions associated with increased risk of perforation include prior bowel surgeries, bowel obstruction/ileus, diverticulitis, and rectovaginal nodularity [14]. Certain types of cancer, including ovarian cancer, also predispose to gastrointestinal perforation. It has been reported that gastrointestinal perforations occur in 15% of patients treated with bevacizumab for ovarian cancer [15]. Treatment-related factors for gastrointestinal perforation include abdominal irradiation, bowel surgery, nonsteroidal anti-inflammatory drugs (NSAIDs), and steroids.

In the present case, neutropenic enterocolitis was probably responsible for increasing the risk of intestinal perforation associated with bevacizumab. Neutropenic enterocolitis occurs as a result of injury to gut mucosal integrity in severely neutropenic patients. It follows treatment with cytotoxic chemotherapy, including carboplatin and paclitaxel [16]. It is however rare, occurring in about 5% of neutropenic patients [17]. Despite the low incidence, it is associated with very high mortality rates, exceeding 50%. Clinically, neutropenic enterocolitis is characterized by fever and abdominal pain, particularly in the right lower quadrant [18]. Additional bacterial invasion of the damaged bowel wall may lead to transmural inflammation and subsequent perforation. Peritoneal signs and shock clinically indicate the possibility of intestinal perforation. CT could be a useful tool in the diagnosis as it adequately demonstrates bowel wall thickening, bowel dilation, and pericolonic inflammation [19]. These findings were all noted in the present case.

Two factors, namely, the administration of bevacizumab and the occurrence of neutropenic enterocolitis, might have synergistically led to gastrointestinal perforation in the present case. The bowel wall might have been initially inflamed and damaged by bacterial invasion resulting from neutropenic enterocolitis; concurrent bowel ischemia induced by bevacizumab probably prevented healing of the intestinal mucosa, contributing to intestinal perforation. Since it is a rare complication, neutropenic enterocolitis has not been recognized as a risk factor for gastrointestinal perforation.

The present patient also had thrombocytopenia of grade 4 on admission. Although severe pancytopenia is rare in patients receiving the gemcitabine/carboplatin plus bevacizumab regimen, it is a severe adverse event [3]. Severe thrombocytopenia is potentially fatal, and surgery is contraindicated in many cases. In the present case, the patient was able to undergo surgery and had recovered gradually from the intestinal perforation despite concurrent pancytopenia of grade 4 and neutropenic enterocolitis.

To the best of our knowledge, this is the first reported case of intestinal perforation resulting from neutropenic enterocolitis in a patient treated with gemcitabine/carboplatin plus bevacizumab for ovarian cancer. Neutropenic enterocolitis is very rare among patients treated with bevacizumab. However, it is necessary to recognize the considerable increase in the risk of bevacizumab-related gastrointestinal perforation in patients having neutropenic enterocolitis consequent to cytotoxic chemotherapy.

In conclusion, bevacizumab in conjunction with neutropenic enterocolitis led to intestinal perforation in this patient with ovarian cancer. Patients undergoing treatment with bevacizumab should be carefully evaluated for concurrent neutropenia. In the event of intestinal perforation, it is essential to institute immediate and appropriate measures for management.

## Figures and Tables

**Table 1:** Laboratory data of posttreatment day 14. Decline of leukocytes, neutrophils, and platelets. Elevation of C-reactive protein. A blood gas analysis did not show any abnormal data.

Laboratory data		
Complete blood count		
WBC	400	/*μ*L
Neutrophil	130	/*μ*L
RBC	2.97	×10^6^/*μ*L
Hb	8.7	g/dL
Hct	25.4	%
Plt	1.0	×10^4^/*μ*L
Blood coagulation test		
PT	108	%
PT-INR	0.97	
APTT	28.3	Seconds
Blood gas analysis		
pH	7.46	
PaO_2_	83.0	mmHg
PaCO_2_	39.3	mmHg
HCO^3^	27.3	mmol/L
BE	3.3	mEq/L
Lactate	16	mg/dL
Blood biochemical test		
TP	5.2	g/dL
Alb	2.3	g/dL
T-bil	0.7	mg/dL
AST	25	IU/L
ALT	23	IU/L
LDH	233	IU/L
CK	24	IU/L
BUN	19	mg/dL
Creatine	0.5	mg/dL
Na	142	mmol/L
K	3.2	mmol/L
Cl	103	mmol/L
Ca	8.2	mg/dL
CRP	16.5	mg/dL
